# 

**Published:** 1856-10

**Authors:** 


### CONTENTS.

ORIGINAL ESSAYS AND COMMUNICATIONS.

The action of Topical Remedies on Inflamed Dentine. By GEO. WATT, ........ 1
Gangrenous Degeneration of the Cheek and Gums, with Necrosis and Exfoliation of the Alveolar Processes, and Maxillary Bone. By J. RICHARDSON, D. D. S., M. D., ........ 16
Gutta Percha, as an application to Clasps. By EDMUND OSMOND, D. D. S., ........ 26
Spirit-Lamp Explosions, ........ 30
Preparation of Plaster Models, ........ 32
Hypertrophy of the Gums, ........ 33
Dental Operations, ........ 34

PROCEEDINGS OF SOCIETIES.

Sixteenth Annual Meeting of the American Society of Dental Surgeons, ........ 37
Report of the Meeting of the Western Dental Society, at Chicago, Ill., in the International Hall, July 30th, 1856, ........ 39
Second Annual Meeting of the American Dental Convention, ........ 63
Editorial, ........ 147
Obituary, ........ 152

